# 'Why Aren't We Fighting Our Case?': Speech and Language Therapists’ Perspectives on Intervention for Preschool Children With Oral Comprehension Difficulties

**DOI:** 10.1111/1460-6984.70112

**Published:** 2025-08-19

**Authors:** Katherine Shobbrook, Rosie Miller, Shybah Yunis, Suzanne Beeke, Wendy Best

**Affiliations:** ^1^ Language and Cognition UCL London UK; ^2^ Imperial College Healthcare NHS Trust London UK; ^3^ St George's NHS Trust London UK

**Keywords:** comprehension, evidence‐based practice, language disorder, preschool

## Abstract

**Background:**

Oral comprehension difficulties are prevalent amongst preschool children with language needs and are related to academic, social and emotional outcomes. Speech and language therapists (SLTs) frequently deliver comprehension intervention to preschool children, but the influences on their clinical decisions are unknown. Understanding these influences and how chosen interventions align with models of evidence‐based practice (EBP), particularly in an area where research evidence is sparse, is crucial to developing effective intervention and supporting the implementation of research to practice.

**Aims:**

To investigate SLTs’ perspectives on the delivery of intervention to preschool children with oral comprehension difficulties and to examine these in the context of models of EBP.

**Methods and Procedures:**

Semi‐structured interviewing gathered the perspectives of 14 UK‐based SLTs, representing a range of years of experience, work settings and employment models (NHS and independent). Data were analysed using reflexive thematic analysis.

**Outcomes and Results:**

The overarching theme ‘Flexibility and Constraint’ described a complex and challenging process whereby SLTs respond to sometimes conflicting influences from external drivers, the needs of the child and adults around them, perspectives on who has responsibility for effecting change, and their own perceptions of clinical autonomy.

**Conclusions and Implications:**

SLTs’ practice aligns with components of EBP, but the need to be flexible and responsive to individual circumstances is frequently in conflict with constraints affecting the ability to deliver individualised intervention. Implications include how individual SLTs and speech and language therapy services promote the ways in which they are evidence‐based practitioners and advocate for their specialist role in supporting preschool children with oral comprehension difficulties.

**WHAT THIS PAPER ADDS:**

*What is already known on this subject*

- Models of EBP emphasise the considered application of research, patient preferences and clinician‐related factors. This process can be a challenge where few research studies reflect the wide variety of approaches used by SLTs, and contextual factors such as organisational culture and funding may take precedence in decision‐making.

*What this paper adds to existing knowledge*

- This study describes a very wide variety of practices, with some elements aligning with the principles of EBP and others deviating from them. The lack of time and resources is a common constraint that affects the ability to deliver individualised intervention and therapy which reflects up‐to‐date research in this area.

*What are the potential or actual clinical implications of this work?*

- Perspectives on clinical autonomy and the need to advocate for the specialist role of the SLT have far‐reaching implications. These include how therapy for individual children with oral comprehension difficulties is planned and delivered, how speech and language therapy services are structured, and how the profession as a whole advocates for its role with preschool children experiencing comprehension difficulties.

## 1 Introduction

Oral comprehension difficulties can involve problems understanding single words, sentences and/or discourse and are often a key feature of language disorder, whereby difficulties with spoken language significantly impact an individual's everyday functioning (Bishop et al. 2017). Children with language disorder may be diagnosed with developmental language disorder (DLD) when language difficulties exist without additional conditions, or language disorder associated with X, where X is a known biomedical aetiology, such as autism or intellectual disability. Language disorder is relatively common, affecting 9.92% of children at school entry (Norbury et al. 2016), equating to up to three children per class. DLD can have significant impacts on educational outcomes, career prospects, participation and wellbeing (Botting et al. 2016; Conti‐Ramsden et al. 2018), and severity of comprehension impairment is associated with long‐term prognosis (Clark et al. 2007; LaParo et al. 2004).

Oral comprehension difficulties in children under 5 years of age are frequently the target of speech and language therapy/pathology (henceforth speech and language therapy) intervention within services to preschool children (using the UK definition of ‘preschool’ as children under the age of 5 years). *Child Talk*, a detailed and large‐scale investigation of clinical practice in the United Kingdom (Roulstone et al. 2015), found that 89% of speech and language therapists (SLTs) surveyed regarded intervention for comprehension as ‘essential’ to their practice with preschool children with developmental speech and language disorders. SLTs reported using a wide range of activities and approaches (41 in total) to address comprehension, which varied from addressing the impairment using structured adult‐led tasks to supporting the child's understanding of the environment using visual support systems. One implication of this range is difficulty developing practice guidelines, which in turn can challenge the implementation of evidence‐based practice (EBP) (Morgan et al. 2019).

EBP is the cornerstone of clinical practice and, accordingly, is central to professional requirements for speech and language therapy. The Health and Care Professions Council (HCPC), the regulatory body for SLTs in the United Kingdom, requires all registrants to engage in EBP (HCPC 2023), and in the United States, the Code of Ethics of the American Speech‐Language‐Hearing Association (ASHA) states that practitioners shall use evidence‐based clinical judgement (ASHA 2023). In these regulations, organisations draw upon the model of EBP developed by Dollaghan (2007), describing the conscientious integration of the best available: (i) research evidence, (ii) practice evidence, and (iii) patient preferences. EBP is portrayed as an active process that requires careful and critical examination of a range of evidence pertinent to each case.

Models describing the process of EBP have been critiqued by a range of researchers examining clinical decision‐making in speech and language therapy. Dollaghan's often‐cited three‐part model, whilst stating the importance of factors relating to the practitioner and their client, has been criticised for an over‐emphasis on research evidence in a profession where research can fail to represent the complexities of intervention for individuals with communication disorders (McCurtin et al. 2019). Furthermore, researchers have argued that the research evidence that is available is often under‐represented in decision‐making. Factors taking precedence are often the clinician's knowledge and experience, characteristics of the client and their needs, or pragmatic considerations such as the availability of resources (Forsythe et al. 2021; McCurtin and Clifford 2015).

Such pragmatic considerations are captured within a further component of EBP: contextual evidence. Initially used in nursing (Thompson et al. 2002), this component is an integral part of Hoffman and colleagues’ contemporary model of EBP for health professions (Hoffmann et al. 2024). Applied to speech and language therapy, Law (2019) calls this component ‘other drivers’ and describes how it encompasses factors such as service funding, the overall culture of an organisation, the influence of significant practitioners, and physical access to services due to geography. Whilst these areas are included within models of EBP, it is noteworthy that such considerations may not always be consistent with best practice. For example, there may be a negative impact of organisational culture, and individuals may be denied services or struggle to access them due to geographical location.

Considering the research evidence for oral comprehension interventions for preschool children, some studies suggest that intervention can be effective (Tarvainen et al. 2020), but there are few studies that reflect typical clinical caseloads (Shobbrook et al. 2024). Knowledge of the theory underpinning interventions can contribute to EBP (McCurtin et al. 2019), and this knowledge may be particularly important where the research evidence is sparse. Law et al. (2008) examined the clinical decision‐making of SLTs working with children 5 to 11 years old with severe oral comprehension impairments. They found that theory was used very infrequently, and when theories were described, they tended to be translational, in the sense that they derived from intervention programmes developed to target a particular deficit, rather than theories of language development or impairment. Similarly, the *Child Talk* report described above stated that there was no unified theory of therapy underpinning SLTs’ decisions for preschool children with speech and language impairment. Many therapists described decisions based upon hierarchical models of language development, such as the so‐called ‘communication pyramid,’ that are unsupported by empirical research and have been critiqued in existing studies of practice (Morgan et al. 2019).

Descriptions of research thus far have focused upon studies of intervention approaches selected by SLTs and their rationales for these decisions. Other studies have taken a different, but complementary angle by investigating SLTs’ perspectives on intervention. Marshall et al. (2007) reported that SLTs working with preschool children with language difficulties commonly viewed their role as providing a facilitative communicative environment for the child. This involved working with families and education settings and was reported by SLTs to have varying levels of success. The perceived importance of the partnership between parents/caregivers and the SLT has been described by other studies (Davies et al. 2019; Klatte et al. 2019; Klatte and Roulstone 2016), where parental engagement was considered central to the success of an intervention. Despite this, SLTs reported that engaging parents was one of the most challenging aspects of intervention with preschool children, and that organisational constraints sometimes prevented the flexibility necessary to adapt practice to individual families' needs.

There have been no studies that specifically explore SLTs’ practice for preschool children with oral comprehension difficulties in depth, despite the prevalence of these needs, the relationship between comprehension and prognosis, and the long‐term consequences of language disorder. The wide number of approaches used in practice versus the small, but growing body of research makes it vital that we understand the factors influencing SLTs’ selection of certain approaches over others. Doing so will support the implementation of research to practice and will enable future research to address areas of concern for practitioners. Examining decision‐making processes can also provide information about how the principles of EBP may be reflected in SLTs’ working lives. Understanding the factors that drive clinical decisions is thus crucial to both developing and delivering the best intervention.

This study aims to investigate the perspectives of SLTs delivering intervention to preschool children with oral comprehension difficulties, and to examine these in the context of models of EBP. It seeks to answer the research question: what influences the practice of SLTs delivering intervention to preschool children with oral comprehension difficulties?

## 2 Methods

This study followed a qualitative research design. Semi‐structured interviews were used to collect the data, as they allowed the researchers to probe participants’ perspectives in depth whilst maintaining focus on the topic. Ethical approval was granted by the UCL Departmental Ethics Committee (LCD‐2021‐06). Participants were recruited over two phases in February 2022 and June 2023 through professional networks of author K.S., social media (Twitter/X) and emails sent to the Association of SLTs in Independent Practice and relevant Royal College of Speech and Language Therapists (RCSLT) Clinical Excellence Networks. Inclusion criteria were that participants were registered SLTs working in the United Kingdom with preschool children.

There were 37 expressions of interest from registered SLTs, 20 of whom agreed to participate and returned a signed copy of the consent form and demographic information. A purposive sampling method was used to select 14 participants representing a range of geographical locations in England, years of experience (1 to 32 years) and employment (independent and NHS) (see Table 1). Participants comprised 13 females and one male; there were no participants from Wales, Scotland or Northern Ireland. Interview duration was an average of 48 min, 18 s (range 38:01–57:33).

**TABLE 1:** Participant characteristics.

Participant	UK location	Years of experience	Service type
1	West Midlands	10	Independent
2	South East	32	Independent
3	London	7	NHS
4	West Midlands	2	NHS
5	South East	12	NHS and Independent
6	South East	5	Independent
7	East of England	5	NHS
8	Yorkshire and the Humber	30	Independent
9	North West	3	NHS
10	South West	15	Independent
11	South East	14	NHS
12	North West	1	NHS
13	East of England	2	NHS
14	London	1	NHS

## 3 Interviews

An interview guide was drafted by authors K.S., R.M. and S.Y. and piloted with two SLTs. This consisted of 12 open questions and a series of probes to elicit further information if necessary. Pilot interviews showed that the guide was effective in eliciting information to address the research question. Minor amendments were made to the wording of three questions/probes, and the order of two questions was reversed to facilitate a more logical flow to the interview. See Appendix for the final interview guide. Interviews were conducted over two time periods which aligned with co‐authors’ research schedules: April and May 2022 (interviewers R.M. and S.Y.; interviewees 1 to 6) and June and July 2023 (interviewer K.S.; interviewees 7 to 14).

Interviews were conducted online via MS Teams and recorded and saved to an encrypted secure drive in accordance with university data management procedures. Transcripts generated by MS Teams were checked and amended following guidelines described by Braun and Clarke (2013). Participants were assigned a pseudonym, and all places and services mentioned were anonymised.

## 4 Data Analysis

K.S. analysed the data using reflexive thematic analysis (RTA) (Braun and Clarke 2006, 2022). K.S. is an SLT with more than 20 years’ clinical experience with children with language difficulties. RTA was appropriate for two reasons: it recognises the values and experience the researcher brings to the process of enquiry and analysis, and it is particularly suited to research attempting to understand people's experiences, views and perspectives.

Data analysis followed a six‐phase process: (1) dataset familiarisation; (2) data coding; (3) initial theme generation; (4) theme development and review; (5) theme refining, defining and naming; and (6) writing up, with the understanding that rather than being strictly linear, this process is recursive and involves moving forward and backwards between phases. Analysis was both inductive, capturing and describing meanings from participants’ experiences and perspectives, and deductive, drawing upon the knowledge and experience of the main researcher and research team, and current (limited) literature. Codes were semantically‐orientated in the early stages of analysis and became more latent and fewer in number in successive rounds, as engagement with the dataset and discussions with co‐authors led to a deeper understanding of the data. This method led to the creation of both inductive and deductive themes and subthemes.

Reflexivity was ensured throughout the process in several ways. Prior to the analysis, K.S. conducted a written reflection of her personal, functional and disciplinary reflexivity and reflexivity around the topic of intervention for preschool children with oral comprehension difficulties (Braun and Clarke 2022). This acknowledged existing assumptions and how they had shaped and informed the research. During the analysis, thoughts about the data and reflections on the overall process were noted in a reflexive research journal. Different methods of interacting with the dataset (working from paper and electronic transcripts during coding; drawing and updating mind maps when developing and refining themes and writing up) and working from different physical locations facilitated the focus necessary for sensitive and deep engagement with the data. Throughout the process, discussions with a colleague highly experienced in RTA but not involved in the research, and with W.B. and S.B., allowed K.S. to reflect on her position as a researcher and clinician and gain greater depth of understanding.

## 5 Results

**FIGURE 1:**
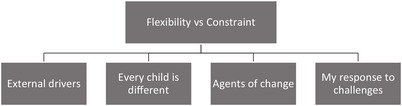
Thematic map.

Analysis identified four subthemes: *External drivers, Every child is different, Agents of change* and *My response to challenges*. *External drivers* and *Agents of change* were derived primarily deductively; *Every child is different*, and *My response to challenges* from inductive analysis. The subthemes are united under the overarching theme *Flexibility* *versus Constraint* (see Figure 1). Flexibility refers to responding and adapting to circumstances related to the child, family and practice context, whilst constraint describes the challenges and limitations participants experience in their work. A description of each subtheme is presented below, with quotes from the transcripts written in italics to preserve the voice of the participants and distinguish them from the narrative. Intervention approaches named by the participants are in bold and listed in Table S1 (Supplementary Material).

## 6 External Drivers

This subtheme describes how decision‐making is informed or influenced by factors external to the child, family and/or environment. These factors are theories of language development and impairment, research evidence, and service pathways and standardised intervention packages, which are often influenced by capacity and resource.

Some participants’ decisions were influenced by the way they understood language and communication to develop in young children, often described as a process of moving through a series of pre‐determined stages or levels. The analogy of a pyramid was used to describe how skills in one area are the foundation for the development of the next. Cognitive skills such as joint and sustained attention, and play were described as foundational to communication development, and this understanding influenced the way that intervention was planned and delivered:

*you think about the communication pyramid, you need all of those fundamental early skills in order to be able to say the right sounds (P7)*

*I also planned the sessions in terms of the communication pyramid. So thinking about making sure that all the foundation levels like attention and play… Moving up the pyramid week by week was my ongoing plan for the therapy sessions (P6)*

Some participants justified their intervention approach in terms of how children develop their understanding of symbolic representation. This was also expressed in terms of the achievement of a series of stages:

*(describing selection of visual resources) So object understanding comes before picture understanding, so obviously it goes object all the way down to line drawings (P7)*

*[objects of reference is] usually what we try first… because it's lower down on that level of symbolic understanding (P3)*

Other participants expressed the view that improvements in comprehension and expression are mutually supportive, and that rather than being a foundation for later language work, work on play can simultaneously lead to ‘*benefits across a whole range of domains*’ (P2).

In terms of theories of language impairment, participants described the necessity for careful assessment. Assessment was linked to the identification of red flags for language disorder or future diagnosis of DLD and was also used to identify the most appropriate therapeutic targets and approach. For the latter, the assessment method and the intervention approach were closely related. Descriptions revealed an iterative process in the selection of assessment and intervention:

*if I was able to do the* ***DLS*** *with her, then I'd be able to specifically say ‘OK, it's this area we need to work on’… So I've had to find one thing that's difficult and say ‘let's just focus on that’. And then after that we'll do some more assessment…and say, ‘OK, now this is difficult. Let's work on that.’ (P4)*

*when I [do the] Preschool CELF I always do that Basic Concepts assessment… That one I always find really useful because if I see gaps in that area then I will give them…a few concepts activities (P13)*

Alternative perspectives involving consideration of the child's communication needs within their environment are described in *Every child is different*.

Research evidence contributed to participants’ intervention decisions, and the phrase ‘*evidence base*’ was widely used in this context. When probed, participants described research findings from peer‐reviewed intervention studies and systematic reviews, and articles in *Bulletin*, the UK SLT professional magazine. Participants also saw the evidence base as comprising resources developed from research findings, such as professional guidance and ‘*toolkits*’ for assessment and intervention.

Participants commented on the lack of research evidence for oral comprehension interventions and the fact that this was a barrier to working in an evidence‐based way. One specifically described difficulties finding evidence to support ‘*decisions based on the child's functionality*’ (P1), whilst another said that the lack of research for frequently used approaches caused her to doubt her decision‐making:

*A lot of the* ***Black Sheep Press*** *stuff*, ***Derbyshire Language Scheme*** *, they're not informed by research. So I always feel when I'm working with children with verbal comprehension difficulties: am I actually doing the right thing? (P2)*

Another external driver for decision‐making was the application of organisational systems such as care pathways and standardised intervention packages. Pathways describe a child's route through a service and are selected on the basis of their language profile or diagnosis:

*we have a clear pathway in terms that once they have done the assessment then there are different packages depending on the child; like diagnosis or the child's profile (P14)*

Intervention packages specified the details of intervention, such as ‘*who does it and when, and how often it happens*’ (P5), as well as the approaches to be used.

In some cases, service pathways were influenced by a tiered model of provision, where only those meeting a ‘*threshold of needing specialist level support*’ (P9) receive intervention from an SLT. Such children were identified through the presence of risk factors and indicators of an ongoing language and/or communication difficulty or had an existing diagnosis of DLD.

The main external driver behind the creation of service pathways and intervention packages was capacity, specifically time and SLT staffing. In some cases, caseload size and scarcity of staff resulted in services delivering only assessment and advice, or where intervention was offered, this was not targeted at oral comprehension:

*because we have just four sessions and we can't just work on receptive language (P14)*

In some cases, the time available was the starting point for clinical decisions and had influenced the decision to select a particular approach on a service‐wide basis:

*I think [the service] picked* ***VERVE*** *because it was four sessions…So they could do it in less time (P13)*

The influence of resourcing constraints was not limited to SLTs working in NHS services. One participant described how intervention delivered in independent practice is often decided from the funds available, a restriction that is not always understood by others:

*in the NHS, they think that in independent practice we can just give masses and masses of therapy and everyone loves us sort of thing, and it's not the case because obviously somebody has to fund it… We are still restricted by what can be paid for. (P8)*

Whilst SLTs in both independent and NHS services reported, at least to some extent, the use of standardised pathways and packages, participants working within the NHS often described being ‘*told*’ to follow certain procedures or reported that these had been ‘*decided*’ by somebody else. This suggests a lack of autonomy in decision‐making which is explored further within *My Response to Challenges*.

## 7 Every Child Is Different

This subtheme describes the child at the centre of decision‐making and includes effects of the physical and social environment on planning and delivery of intervention.

The starting point for many participants was a consideration of the priorities of both the child and family. This included spending time getting to know the child, identifying what is meaningful and important to them, and planning intervention to increase the child's participation in their environment. Because participants acknowledged that every child is different, some described feeling constrained by care pathways or were critical of the decisions based solely upon frameworks or programmes:

*I think sometimes we can get really caught up with norms and data and we forget about the individual and what is going to increase their function. (P1)*

*I do tend to lean far more into that functional overview approach of what does the child need, versus what package can I fit to them (P11)*

This subtheme encompasses the fact that the parents, carers and education staff around each child are different. Participants commented on the wide variety of people's knowledge, skills and capacity, resulting in the need to tailor intervention to individual circumstances. Working with families to develop a realistic intervention plan around their capacity was viewed as important:

*[I] say ‘you know, what is actually feasible?… It's more so that I know how things work and I can help work out what's gonna work for you rather than me critiquing you and saying ʻwhy didn't you manage to find someone to babysit that child?ʻ’ (P5)*

The differing needs of the people around a child sometimes created tensions in services offering the same number of sessions for all families:

*I got more frustrated by the policy of six‐week blocks because I don't necessary think that that's the right thing for all families (P5)*

One participant, setting up an independent service after many years in the NHS, described the creation of a flexible service starting from the existing skills of those in the child's environment:

*I think it depends on what's already in place, cause some children are in settings that are…doing lots of things… We don't really…have a care pathway that says ‘if the child has this difficulty, this is what they get’…and I think that's a strength really (P8)*

## 8 Agents of Change

*Agents of change* describes perspectives on the role of the SLT and people around the child: parents and carers, nursery/preschool teachers and support staff. Divergent views were expressed within this theme, highlighting differences in the way that participants saw their own and others’ roles in effecting change in the child's communication.

Whilst participants expressed the view that intervention involves supporting other people to develop the child's language, participants had different views on how this is achieved. For participants working within services providing assessment and advice as a result of capacity restrictions, their role involved advising and showing others what to do:

*it's more consultative really, sort of agreeing targets with parents and nurseries… showing them how to implement the therapy (P12)*

*I'd talk them through some of the strategies that you would use…and I would demonstrate that with them, and then I'd ask them to give it a go (P13)*

Those working in services providing intervention with greater regularity described a more flexible and individualised approach, often focused on helping parents to understand how and why to use an intervention. This included the benefit of prioritising time at the beginning of a period of intervention:

*I've had some recent experiences where we've put some more time in earlier on…and you kind of see that light bulb moment where the parent's like ‘I've got it now, I understand!’… And then feeling more confident they will go away and really embed that into their day (P9)*

A key divergence within *Agents of change* was around perspectives on the ultimate responsibility for effecting change in children's communication. For participants working within consultative services or those providing indirect models of therapy, the outcome of the SLT's input was the training of others to deliver the intervention to the child: the trained person being the agent of change. One participant expressed the benefits of this model:

*I think it's positive to promote that you don't have to be a speech therapist to work on something specifically, and it emphasises…it's kind of all of our job to support a child's communication (P7)*

Another participant felt that when working with children with oral comprehension difficulties, developing others’ skills replaced the need for intervention from an SLT:

*I would rarely see a child one to one if they had any receptive language difficulties, it would usually be working through the parents, nursery, upskilling them… (P9)*

These standpoints were not shared by all participants. For some, children with language difficulties develop their skills through direct therapy with an SLT: the SLT is the agent of change. An argument directed against consultative and indirect models was the loss of the SLT's specialist knowledge and expertise. Participants described the acquisition of expertise over time, occurring through learning from experience, discussions with colleagues and engagement with relevant literature and research. Some participants who had been working for more than 5 years described making decisions intuitively as a result of their experience. Many talked about how their practice had been enhanced over the years, particularly in becoming more responsive and tailored to child and family backgrounds and circumstances:

*I rarely now as a more experienced therapist, I rarely would just pull out a ready‐made little set of pictures (P2)*

*because I have worked with such a variety of cultures, I have adapted and built up my confidence in explaining the therapy that's being offered… that experience has helped me refine my speech that I give to parents to help best support their understanding of what their therapy is. (P6)*

Receiving training in particular approaches was an influential factor in the decision to select them over others; however, participants experienced constraints in accessing training, which had an impact on the effectiveness of the intervention. This viewpoint was explained by one participant who reflected on the tensions faced by working in a service that requires non‐specialists to deliver approaches designed to be delivered by SLTs:

*I find it's hard to be able to get training…in a range of different interventions, but you're expected to deliver them… We're asking someone to deliver something which is speech therapist‐led but I've never been on the training for that… I'm asking someone else to do it too when I'm not fully, completely sure on what it is ((laughs)) at times (P7)*

Two participants, amongst the most experienced in the group, gave their views on the reason for the increasing use of indirect or collaborative services. They described it as a response to lack of capacity and felt that emphasising the role of others had resulted in devaluing the SLT's specialist skills. They talked about the collective responsibility of SLTs for the loss of their direct role:

*As a profession we've bought into anybody can do speech therapy, and I don't think that's true… We've given that up too easily (P8)*

*we are constantly devaluing ourselves because the NHS is so incredibly stretched. We say, ‘Oh, it's fine. You don't need us…you don't need a therapist…we're going to discharge you, the nursery staff can do it.’ (P1)*

One participant strongly argued for the need to advocate for the specialist role of the SLT for children with language disorders and the necessity of doing so to protect the profession:

*we're specialists for a reason… We should be shouting ‘No, you know what? This person does need a speech and language therapist. This child has a receptive language disorder… This isn't something a teaching assistant or a nursery key worker can do’… They need us. That's why we're here as a profession… Why aren't we fighting our case a little bit more? (P1)*

Despite strong opinions on the necessity for SLT‐delivered intervention, participants felt that direct work still needed the input of parents/carers and education staff:

*[I] don't feel if I did it all and no one else did anything, it would work either (P12)*

To this end, participants described the importance of teamwork, involving shared decision‐making based upon mutual understanding, negotiation and compromise. Participants felt that the success of working with others depended upon a good knowledge of the child, family and setting, and that regular and frequent contact with the same SLT was important for forming relationships and establishing trust.

## 9 My Response to Challenges

This subtheme presents descriptions of how participants respond to constraints and other challenges. It includes reflections and actions in response to the difficult aspects of clinical decision‐making and perspectives of their own autonomy in the face of such difficulties.

A frequently cited challenge was lack of time, which affected the ability to carry out tasks participants felt they should be doing. This included keeping up to date with research:

*I try to keep up to date with [research] and I try to read [the professional magazine] but I find it is hard with the restraints of the job and the amount of paperwork that we have. It's hard to fit everything in that I would like to. And you feel very drained by the end of the day (P7)*

*Truthfully, [I read research] probably not as much as we should…because of time and you know, demand, and all the rest of it (P8)*

Constraints of time and resources highlighted a tension between what was regarded as the ‘ideal’ versus the reality of clinical practice. Participants responded differently to this challenge. Some described making clinical decisions based on what they were told to do, even when they felt that this was not the right course of action:

*I'm a little bit worried about moving to a monitor and review model for a lot of children…it's what we have to do rather than… what's ideal and what the children necessarily need. That's the situation we've been pushed into. (P3)*

*Sometimes, it doesn't feel like you can do what clinically you should be doing because you…have to follow what we can offer or what you're being told from managers (P7)*

Other participants exercised autonomy within the constraints of the system where they could. This included the conscious decision to take a more flexible approach to better meet the needs of individual children or families:

*I am more flexible in my approach and I sort of argue the rationale if I'm questioned (P5)*

*I would be giving slightly tweaked advice and that was my on my own decision to do that because I knew that it needed to be slightly different (P13)*

In basing decisions on what was pragmatic for each situation, some participants reflected that some of their actions were not supported by research or guidelines. This working in ‘*clinical reality ways*’ (P2) included how they work with interpreters, make decisions about the duration of treatment, and, as described below, how training is implemented in practice:

*I went to the* ***Colourful Semantics*** *training for example and they have a way that they want that to be done, but then I have to adapt that to what we're doing, what we have time to do… At the training, I said, ‘you know I've got to bring what you said and do it in a block of five sessions and then the TA's got to continue doing it. How do I do that effectively?’. And they were like, ‘you can't’. And I was like, ‘well’ ((laughter)), ‘well, I do have to. Somehow.’ (P13)*

The challenges of time and resources resulted in participants often feeling *‘frustrated*’ by the service they deliver or had delivered in the past. Commonly expressed views were that children with language difficulties were not seen early enough, intervention delivered by others was not followed up frequently enough, and direct intervention from an SLT was either not available or not given in sufficient quantity or regularity. Participants 1 and 2 working in independent settings, by contrast, used the word ‘*luxury*’ to describe the time they had to work with children and families, acknowledging that this was not the norm.

Participants described delivering intervention as something they enjoyed. Delivering therapy made them ‘*happy*’ (P11), was ‘*exciting*’ (P9) and was a part of the job that ‘*I love*’ (P10). These positive feelings were related to the belief that direct intervention was effective; that in delivering therapy and seeing the progress made by children and their families, they were doing something that made a difference.

## 10 Discussion

This study set out to explore the factors that influence SLTs’ clinical decision‐making for preschool children with oral comprehension difficulties. The overall theme of *Flexibility versus Constraint* and subthemes suggest that decision‐making in this context is a complex and challenging process whereby clinicians negotiate a path through conflicting and sometimes contradictory influences from external drivers, the needs of the child and the adults around them, and perspectives on who has the ultimate responsibility for effecting change in the child's communication. In doing so, participants described varying responses to these challenges, providing insights into how they regard their clinical autonomy and, ultimately, how they engage in EBP. The findings of this study contribute to knowledge regarding how SLTs interpret and use the principles of EBP, and support research describing the precedence of context (Forsythe et al. 2021; McCurtin and Clifford 2015) and the challenges of offering flexible, child‐led services (Klatte and Roulstone 2016).

## 11 Flexibility and Constraint

A central tension identified through the analysis is the provision of flexible and individualised intervention, where goals, approaches and delivery are tailored to each child and family, versus the use of pathways and standardised intervention packages, developed in response to the constraints imposed by lack of resources, most commonly time. Whilst some participants described providing flexible and responsive intervention, for others, resource limitations resulted in difficulties delivering intervention at the timeliness, intensity or duration they felt was required, or that matched the dosage used within research studies. One participant gave the lack of time as the reason for not addressing oral comprehension at all. These findings are significant when considered alongside the 89% of SLTs reporting that comprehension interventions are essential to their practice (Roulstone et al. 2015).

Research evidence supports intervention for children with DLD (Ebbels et al. 2019) and the effectiveness of intervention for oral comprehension in preschool children with language difficulties (Shobbrook et al. 2024; Tarvainen et al. 2020). Therefore, the lack of individualised, direct intervention for children with oral comprehension difficulties is concerning and may have long‐term consequences on their language development. Furthermore, planning services from the standpoint of the availability of resources and offering a set duration for all children goes against the recommendations of the professional body for SLTs in the United Kingdom, the RCSLT, who state that:

*Models of service delivery that are based on blocks of time or numbers of sessions are not supported by evidence and do not represent best practice… Speech and language therapy advice must address the child or young person's assessed needs rather than the availability of resources*.

*Position Statement: ‘Caseload management of children's services’* (RCSLT n.d)

The frustrations, worries and impact on the enjoyment of work when SLTs were not able to deliver intervention are highly significant when considered alongside the relationship between job satisfaction and retention. At the time of writing, there is a reported vacancy rate of 25% in children's services across England (RCSLT 2024a), an alarming amount considering the estimated 1.9 million children in the United Kingdom with speech, language and communication needs (Speech and Language UK n.d.). Results from this study corroborate findings in other areas of speech and language therapy practice that lack of time to deliver services impacts job satisfaction and therefore may have a role in the current staffing crisis (Hutchins et al. 2010).

Given the shortage of SLTs, it is understandable that services have adapted to use SLTs’ time and skills for the training and development of others (Law et al. 2017, 2002). However, for some participants in this study, this approach risks devaluing their specialist contribution. The implied assertion that ‘*anybody can do speech therapy*’ fails to take into account SLTs’ specialist knowledge and skills, and the value SLTs place upon professional‐led intervention (Pring et al. 2012). Of particular interest is the view that the profession itself bears some responsibility for this position, and there is a need for SLTs to fight to demonstrate their contribution to supporting children with oral comprehension difficulties.

How do SLTs respond to these challenges? Variations were found in this study: participants who went along with services they did not agree with, those who rallied against them, and those who made the changes they could within the constraints of the system. These differing responses reflect varying perspectives on clinical autonomy. Clinical (or professional) autonomy is an individual's participation in decision‐making and their ability to influence working practices and has been found to be related to satisfaction in the workplace and therefore the recruitment and retention of a skilled workforce (Pursio et al. 2021). Taking active steps to encourage SLTs to exercise their clinical autonomy may therefore have an impact on workforce retention.

Clinical autonomy is not simply important for job satisfaction, however; it is a core professional competency. The HCPC emphasises personal responsibility, stating that SLTs must:

*recognise that they are personally responsible for, and must be able to justify, their decisions and actions (Standards of Proficiency: 4:1 (2023))*

The finding in this study that some participants deliver services with which they disagree or are uncomfortable suggests that they do not always exercise this personal responsibility and supports the view expressed by Gallagher (2015) that SLTs may have ‘lost touch’ with their clinical autonomy (57). Making clinical decisions on the basis of ‘the rules’ set by others also has implications on the overall practice of EBP, given that this is an active process requiring ‘reflection and critical thinking’ (McCracken and Marsh 2008, 301).

## 12 EBP

Viewing participants’ perspectives of intervention through the lens of EBP showed a great variety in how its components are reflected in clinical practice with preschool children with comprehension difficulties. Some elements of practice closely aligned with EBP principles, whilst others deviated from what may be considered best practice.

It is noteworthy that when asked about the ‘evidence base,’ participants interpreted this solely in terms of research evidence, and many felt that their use of evidence was lacking due to limitations of time. Despite this, approaches used did often reflect relevant research, such as the use of parent‐based interventions (Roberts and Kaiser 2011), prioritising the child's participation in their communication environment (Baylor and Darling‐White 2020) and using indicators of DLD to prioritise children for direct intervention (Bishop et al. 2016).

The influence of theory was another area where practice deviated from the best available evidence. Similarly to previous studies (Law et al. 2008; Morgan et al. 2019), participants referred to translational theories, hierarchical systems of development and the perspective that certain skills are prerequisites for comprehension. These assertions are unsupported by research and have been discussed in a variety of publications. The RCSLT Clinical Guidelines in Augmentative and Alternative Communication (RCSLT 2024b) urge caution if adopting a hierarchical view of symbolic representation, citing research indicating that it fails to take clients’ individual skills and circumstances into account. The lack of research evidence for the ‘pyramid’ view of communication development was discussed in a peer‐reviewed article (Morgan et al. 2019) and the RCSLT *Bulletin* (Morgan and Dipper 2018), widely read by its 20 000 SLT members. It is possible, therefore, that a consequence of the lack of time to engage with research causes a continuation of beliefs that have been long since questioned within the profession.

Although participants interpreted the ‘evidence base’ as involving only research evidence, their descriptions of intervention decisions showed that they were, albeit without the explicit knowledge of doing so, involving all components within models of EBP. The principles of individualisation and supporting participation‐focused outcomes align closely with patient preferences, and reference to their own expertise and the knowledge gained through experience, training and learning from others correlates with practice knowledge. The fact that these areas had more influence than research evidence supports previous findings on their precedence in clinical decision‐making (Forsythe et al. 2021; McCurtin and Clifford 2015).

Pragmatic considerations were hugely influential for the participants in this study, and many of these aligned with the contextual factors described within models of EBP. Again, there were both positive and negative influences: some participants describing the positive influence of their workplace context and culture, and others describing how the wider context of their work constrained the delivery of best practice. An aspect unexplored by this study is the impact these differences have upon outcomes for preschool children with oral comprehension difficulties, an important area for future research.

## 13 Implications

Influences on decision‐making described in this study map to each component of EBP, providing evidence that participants do engage in EBP in their intervention for preschool children with oral comprehension difficulties. However, this knowledge was not explicit, as participants did not interpret consideration of child‐ and family‐based factors and their own experience and expertise as elements of being an evidence‐based practitioner. ‘*Fighting our case*’ could therefore begin with the explicit assertion of the variety of ways in which SLTs are evidence‐based practitioners.

This study highlights the role of individual responsibility in clinical decision‐making and describes where this may be lacking in practice for preschool children with oral comprehension difficulties. The finding that some participants are delivering services they believe to be contrary to best practice suggests that an additional way of ‘*fighting our case*’ could involve regaining the clinical autonomy and critical judgement that is central to EBP and the practice of speech and language therapy more widely.

Identifying and describing the constraints arising from the workplace context highlights that individual responsibility is not the only issue. This study adds to research (Braithwaite et al. 2018; Selin et al. 2022) showing that SLTs operate within complex systems where beliefs and decisions are often influenced by the conditions and culture of their workplace. Making changes to individual decisions, therefore, involves addressing all parts of this complex system (Selin et al. 2022). ‘*Fighting our case*’ is therefore also an organisational concern. It is vital to create an environment where the specialist contribution of speech and language therapy is recognised and where SLTs can deliver effective and impactful services to children with oral comprehension difficulties.

## 14 Limitations

This study described the experiences of 14 individuals drawn from a range of locations in England, years of experience and employment settings. As such, viewpoints may not be representative of all SLTs working with this population. However, participants were selected to represent as closely as possible the distribution of the UK SLT workforce. The presence of one male was in line with the disproportionate representation of females, and the greater number of participants working in NHS versus independent settings reflects workforce trends across the United Kingdom (HCPC 2021). Furthermore, the process of RTA enabled an identification of shared meanings in the data, and steps taken to ensure reflexivity and a robust approach to data preparation and analysis ensured the validity of the results.

## 15 Conclusions

The variety of influences on decision‐making identified in this study map to the components of EBP: research evidence, practice evidence, patient preferences and contextual factors. However, participants’ explanations of their decisions indicate that this is a complex area, where constraints can affect the effectiveness of intervention and their job satisfaction. Findings have consequences for how individuals regard and practice EBP, particularly relating to individual responsibility. There are implications for how organisations, and the SLTs within them, advocate for the specialist role of the SLT in supporting preschool children with oral comprehension difficulties.

## Supporting information




**Supporting Table S1**: Description of intervention approaches, techniques and resources named by participants

## Interview Guide

**Bold**: Open question

*Italic*: Probe to elicit further information, if necessary

1. **Why don't we start by you telling me about yourself, such as your career as an SLT so far and where you work now?**
2. **Can you tell me about your current role?**

*Who is your employer, how is your service funded, what band are you working at (if relevant)?*

*In which setting/s do you work?*

*What is your role in that setting and in the team?*

1. **Can you tell me about your experience of working with preschool children with receptive language difficulties?**

*For how long have you been working with preschool children with receptive language difficulties?*

*Are there many children with receptive language difficulties on your current caseload?*

1. **Now, I'd like you to think about a preschool child on your current caseload that has receptive language difficulties. Can you describe the child and your involvement?**

*What is the age of the child and profile of their speech, language and communication needs? What setting are they in? Who are the other people involved?*

*What was your role in the process?*

1. **For this child, can you tell me about the stages you went through in the process of planning and delivering their intervention—if you did deliver therapy to them?**

*Did this include delivering therapy, and if not, why not?*

*What were the targets/objectives you set for this child?*

*How did you decide how you would work with the child, such as working individually or in a group, working with parents/others, how often you would see them, where you would see them?*

*How did you decide what intervention approaches or techniques to use? What materials did you use?*

*Did you have to make any adaptations or modifications along the way? If so, what were they and why?*

1. **Do you think that the intervention worked for the child?**

*How do you know? OR What makes you think it didn't work well?*

*Was the therapy carried out as you intended? Do you know?*

1. **How did you feel about the intervention you did (or didn't) provide?**
2. **Now, we're going to think more generally about children on your caseload with receptive language difficulties. What are the factors you consider when planning their therapy?**

*How do you decide what therapy you will deliver?*

*How do you decide what form the therapy will take and the approaches/techniques you'll use?*

*How do you individualise your intervention to the child and their families?*

1. **Again, thinking generally about children that you work with, can you describe the types of therapy you deliver?**

*Who delivers this therapy? Where does it take place?*

*How often does it take place, how many sessions and how long are they?*

*What approaches, techniques or strategies do you use? (If necessary, can you describe them?)*

*What materials do you use?*

1. **How do you choose certain approaches or methods over others?**

*If they mention ‘evidence base,’ ask what they mean by this, for example, What does the expression the ‘evidence‐base’ mean to you?*

*Do you use published research to help you to make decisions?*

*Does the child's environment make a difference? How about other factors related to the child and their family?*

*How does your own experience or that of others around you have an impact?*

1. **Do you think that the interventions you deliver are effective?**

*How do you know: Do you use specific methods to measure your outcomes?*

*Do you monitor whether the intervention is delivered as you intended?*

*Is there anything that particularly supports the intervention working well or not well?*

1. **And now thinking more overall, how do you feel about the service that you deliver to children with receptive language difficulties?**

*What works well?*

*What could be improved?*

*Is there anything that gets in the way of the service you deliver or wish to deliver?*

1. **That's everything I had to ask you to talk about. Have you got anything else you'd like to say or any final thoughts or things you'd like to follow up on that I haven't asked you?**

## References

[jlcd70112-bib-0001] American Speech‐Language‐Hearing Association . 2023. “Code of Ethics.” ASHA. https://www.asha.org/policy/code‐of‐ethics/. 6344876

[jlcd70112-bib-0002] Baylor, C. , and M. Darling‐White . 2020. “Achieving Participation‐Focused Intervention Through Shared Decision Making: Proposal of an Age‐and Disorder‐Generic Framework.” American Journal of Speech‐Language Pathology 29, no. 3: 1335–1360. 10.1044/2020_AJSLP-19-00043. 32463702 PMC7893522

[jlcd70112-bib-0003] Bishop, D. , M. Snowling , P. Thompson , T. Greenhalgh , and the CATALISE‐2 Consortium . 2017. “Phase 2 of CATALISE: A Multinational and Multidisciplinary Delphi Consensus Study of Problems With Language Development: Terminology.” Journal of Child Psychology and Psychiatry 58, no. 10: 1068–1080. 10.1111/jcpp.12721. 28369935 PMC5638113

[jlcd70112-bib-0004] Bishop, D. V. M. , M. J. Snowling , P. A. Thompson , and T. Greenhalgh . 2016. “CATALISE: A Multinational and Multidisciplinary Delphi Consensus Study. Identifying Language Impairments in Children.” PLoS ONE 11, no. 12: e0158753. 10.1371/journal.pone.0168066. 27392128 PMC4938414

[jlcd70112-bib-0005] Botting, N. , K. Durkin , U. Toseeb , A. Pickles , and G. Conti‐Ramsden . 2016. “Emotional Health, Support, and Self‐Efficacy in Young Adults With a History of Language Impairment.” British Journal of Developmental Psychology 34, no. 4: 538–554. 10.1111/bjdp.12148. 27226087 PMC5082521

[jlcd70112-bib-0006] Braithwaite, J. , K. Churruca , J. C. Long , L. A. Ellis , and J. Herkes . 2018. “When Complexity Science Meets Implementation Science: A Theoretical and Empirical Analysis of Systems Change.” BMC Medicine 16, no. 1: 63. 10.1186/s12916-018-1057-z. 29706132 PMC5925847

[jlcd70112-bib-0007] Braun, V. , and V. Clarke . 2006. “Using Thematic Analysis in Psychology.” Qualitative Research in Psychology 3, no. 2: 77–101. 10.1191/1478088706qp063oa.

[jlcd70112-bib-0045] Braun, V. , and V. Clarke . 2013. Successful qualitative research: a practical guide for beginners. SAGE.

[jlcd70112-bib-0008] Braun, V. , and V. Clarke . 2022. Thematic Analysis: A Practical Guide. SAGE Publications Ltd.

[jlcd70112-bib-0009] Clark, A. , A. O'Hare , J. Watson , et al. 2007. “Severe Receptive Language Disorder in Childhood—Familial Aspects and Long‐Term Outcomes: Results From a Scottish Study.” Archives of Disease in Childhood 92, no. 7: 614–619. 10.1136/adc.2006.101758. 17405857 PMC2083799

[jlcd70112-bib-0010] Conti‐Ramsden, G. , K. Durkin , U. Toseeb , N. Botting , and A. Pickles . 2018. “Education and Employment Outcomes of Young Adults With a History of Developmental Language Disorder.” International Journal of Language & Communication Disorders 53, no. 2: 237–255. 10.1111/1460-6984.12338. 29139196 PMC5873379

[jlcd70112-bib-0011] Davies, K. E. , J. Marshall , L. J. E. Brown , and J. Goldbart . 2019. “SLTs' Conceptions About Their Own and Parents' Roles During Intervention With Preschool Children.” International Journal of Language & Communication Disorders 54, no. 4: 596–605. 10.1111/1460-6984.12462. 30784166

[jlcd70112-bib-0012] Dollaghan, C. A. 2007. The Handbook for Evidence‐based Practice in Communication Disorders. Paul H. Brookes Publishing Co.

[jlcd70112-bib-0013] Ebbels, S. H. , E. McCartney , V. Slonims , J. E. Dockrell , and C. F. Norbury . 2019. “Evidence‐Based Pathways to Intervention for Children With Language Disorders.” International Journal of Language & Communication Disorders 54, no. 1: 3–19. 10.1111/1460-6984.12387. 29696726

[jlcd70112-bib-0014] Forsythe, R. , C.‐A. Murphy , J. Tulip , and J. Law . 2021. “Why Clinicians Choose Their Language Intervention Approach: an International Perspective on Intervention for Children With Developmental Language Disorder.” Folia Phoniatrica Et Logopaedica 73, no. 6: 537–551. 10.1159/000513242. 33508820

[jlcd70112-bib-0015] Gallagher, A. 2015. “To Intervene or Not to Intervene: An Exploration of the Conflicts and Dilemmas for the Paediatric Speech and Language Therapist in Practice.” In Speech and Language Therapy and Professional Identity: Challenging Received Wisdom, edited by J. Stokes , and M. McCormick , 43–64. J&R Press.

[jlcd70112-bib-0016] HCPC . 2021. "Diversity Data Report: Speech and Language Therapists." HCPC. https://www.hcpc‐uk.org/globalassets/resources/reports/hcpc‐diversity‐data‐report‐2021.pdf?v=637689354700000000.

[jlcd70112-bib-0017] Health and Care Professions Council . 2023. “The Standards of Proficiency for Speech and Language Therapists.” HCPC. https://www.hcpc‐uk.org/standards/standards‐of‐proficiency/speech‐and‐language‐therapists/.

[jlcd70112-bib-0018] Hoffmann, T. , S. Bennett , and C. Del Mar . 2024. Evidence‐Based Practice Across the Health Professions. 4th ed. Elsevier.

[jlcd70112-bib-0019] Hutchins, T. L. , M. Howard , P. A. Prelock , and G. Belin . 2010. “Retention of School‐Based SLPs: Relationships Among Caseload Size, Workload Satisfaction, Job Satisfaction, and Best Practice.” Communication Disorders Quarterly 31, no. 3: 139–154. 10.1177/1525740109336870.

[jlcd70112-bib-0020] Klatte, I. S. , S. Harding , and S. Roulstone . 2019. “Speech and Language Therapists' Views on Parents' Engagement in Parent–Child Interaction Therapy (PCIT).” International Journal of Language & Communication Disorders 54, no. 4: 553–564. 10.1111/1460-6984.12459. 30729613

[jlcd70112-bib-0021] Klatte, I. S. , and S. Roulstone . 2016. “The Practical Side of Working With Parent–Child Interaction Therapy With Preschool Children With Language Impairments.” Child Language Teaching and Therapy 32, no. 3: 345–359. 10.1177/0265659016641999.

[jlcd70112-bib-0022] LaParo, K. M. , L. Justice , L. E. Skibbe , and R. C. Pianta . 2004. “Relations Among Maternal, Child, and Demographic Factors and the Persistence of Preschool Language Impairment.” American Journal of Speech‐Language Pathology 13, no. 4: 291–303. 10.1044/1058-0360(2004/030). 15719896

[jlcd70112-bib-0023] Law, J. 2019. “Evidence‐Based Practice and Its Application to Developmental Language Disorders.” In Managing Children With Developmental Language Disorder, edited by J. Law , C. McKean , C. A. Murphy , and E. Thordardottir , 6–29. Routledge. 10.4324/9780429455308-2.

[jlcd70112-bib-0024] Law, J. , C. Campbell , S. Roulstone , C. Adams , and J. Boyle . 2008. “Mapping Practice Onto Theory: The Speech and Language Practitioner's Construction of Receptive Language Impairment.” International Journal of Language & Communication Disorders 43, no. 3: 245–263. http://pesquisa.bvsalud.org/portal/resource/es/mdl‐17852535. 17852535 10.1080/13682820701489717

[jlcd70112-bib-0025] Law, J. , J. A. Dennis , and J. J. V. Charlton . 2017. “Speech and Language Therapy Interventions for Children With Primary Speech and/or Language Disorders.” Cochrane Database of Systematic Reviews 2017, no. 1: CD012490. 10.1002/14651858.CD012490. PMC8407295 12918003

[jlcd70112-bib-0026] Law, J. , G. Lindsay , N. Peacey , et al. 2002. “Consultation as a Model for Providing Speech and Language Therapy in Schools: A Panacea or One Step Too Far?” Child Language Teaching and Therapy 18, no. 2: 145–163. 10.1191/0265659002ct232oa.

[jlcd70112-bib-0027] Marshall, J. , J. Goldbart , and J. Phillips . 2007. “Parents' and Speech and Language Therapists' Explanatory Models of Language Development, Language Delay and Intervention.” International Journal of Language & Communication Disorders 42, no. 5: 533–555. 10.1080/13682820601053753. 17729145

[jlcd70112-bib-0028] McCracken, S. G. , and J. C. Marsh . 2008. “Practitioner Expertise in Evidence‐Based Practice Decision Making.” Research on Social Work Practice 18, no. 4: 301–310. 10.1177/1049731507308143.

[jlcd70112-bib-0029] McCurtin, A. , and A. M. Clifford . 2015. “What Are the Primary Influences on Treatment Decisions? How Does This Reflect on Evidence‐Based Practice? Indications From the Discipline of Speech and Language Therapy.” Journal of Evaluation in Clinical Practice 21, no. 6: 1178–1189. 10.1111/jep.12385. 26032767

[jlcd70112-bib-0030] McCurtin, A. , C.‐A. Murphy , and H. Roddam . 2019. “Moving Beyond Traditional Understandings of Evidence‐Based Practice: A Total Evidence and Knowledge Approach (TEKA) to Treatment Evaluation and Clinical Decision Making in Speech‐Language Pathology.” Seminars in Speech and Language 40, no. 5: 370–393. 10.1055/s-0039-1694996. 31426104

[jlcd70112-bib-0031] Morgan, L. , J. Marshall , S. Harding , et al. 2019. “It Depends': Characterizing Speech and Language Therapy for Preschool Children With Developmental Speech and Language Disorders.” International Journal of Language and Communication Disorders 54, no. 6: 954–970. 10.1111/1460-6984.12498. 31531914 PMC6899730

[jlcd70112-bib-0032] Morgan, S. , and L. Dipper . 2018. “Is the Communication Pyramid a Useful Model of Language Development?” RCSLT Bulletin (May, 26 – 28). https://www.rcslt.org/wp‐content/uploads/media/Project/Bulletins/May‐2018———AtE.pdf?la=en&hash=752F7EC88E9A2A3A4013745E321CCD6800FBA283.

[jlcd70112-bib-0033] Norbury, C. F. , D. Gooch , C. Wray , et al. 2016. “The Impact of Nonverbal Ability on Prevalence and Clinical Presentation of Language Disorder: Evidence From a Population Study.” Journal of Child Psychology and Psychiatry 57, no. 11: 1247–1257. 10.1111/jcpp.12573. 27184709 PMC5082564

[jlcd70112-bib-0046] Pring, T. , E. Flood , B. Dodd , & V. Joffe . 2012. “The working practices and clinical experiences of paediatric speech and language therapists: a national UK survey.” International Journal of Language & Communication Disorders 47, no. 6: 696–708. 10.1111/j.1460.6984.2012.00177. 23121528

[jlcd70112-bib-0034] Pursio, K. , P. Kankkunen , E. Sanner‐Stiehr , and T. Kvist . 2021. “Professional Autonomy in Nursing: An Integrative Review.” Journal of Nursing Management 29, no. 6: 1565–1577. 10.1111/jonm.13282. 33548098

[jlcd70112-bib-0035] RCSLT . n.d. “Position Statement: Caseload Management in Children's Services.” RCSLT, retrieved October 25. https://www.rcslt.org/wp‐content/uploads/media/Project/RCSLT/caseload‐management.pdf?la=en&hash=0473471C0912053E044422058877CE549B5C839D.

[jlcd70112-bib-0036] RCSLT . 2024a. “Vacancy Rates in Speech Language Therapy Remain Troublingly High.” RCSLT. https://www.rcslt.org/news/vacancy‐rates‐in‐speech‐and‐language‐therapy‐remain‐troublingly‐high‐at‐21/#:~:text=The%20vacancy%20rate%20in%20adult%20services%20is%2023%25,Yorkshire%2C%20the%20Midlands%20and%20the%20East%20of%20England.

[jlcd70112-bib-0037] RCSLT . 2024b. “Augmentative and Alternative Communication (AAC)—Guidance.” RCSLT. https://www.rcslt.org/members/clinical‐guidance/augmentative‐and‐alternative‐communication/augmentative‐and‐alternative‐communication‐guidance/.

[jlcd70112-bib-0038] Roberts, M. Y. , and A. P. Kaiser . 2011. “The Effectiveness of Parent‐Implemented Language Interventions: A Meta‐Analysis.” American Journal of Speech‐Language Pathology 20, no. 3: 180–199. 10.1044/1058-0360(2011/10-0055). 21478280

[jlcd70112-bib-0039] Roulstone, S. E. , J. E. Marshall , G. G. Powell , et al. 2015. Evidence‐based intervention for preschool children with primary speech and language impairments: Child Talk ‐ an exploratory mixed‐methods study. Southampton (UK): NIHR Journals Library (Programme Grants for Applied Research, No. 3.5) Child Talk phase 1. Available from: https://www.ncbi.nlm.nih.gov/books/NBK311209/. 26312364

[jlcd70112-bib-0040] Selin, C. M. , M. L. Rice , T. M. Girolamo , and C. J. Wang . 2022. “Work Setting Effects on Speech‐Language Pathology Practice: Implications for Identification of Children With Specific Language Impairment.” American Journal of Speech‐Language Pathology 31, no. 2: 854–880. 10.1044/2021_AJSLP-21-00024. 35120298 PMC9150684

[jlcd70112-bib-0041] Shobbrook, K. , P. Young , S. Beeke , and W. Best . 2024. “Making Oral Comprehension Interventions TIDieR: A Narrative Synthesis of Interventions Improving Comprehension in Children From 1 to 5 Years With Language Difficulties.” International Journal of Language & Communication Disorders 59, no. 4: 1351–1370. 10.1111/1460-6984.12998. 38189106

[jlcd70112-bib-0042] Speech and Language UK . n.d. “Speech and Language UK.” Speech and Language UK, retrieved October 25. https://speechandlanguage.org.uk/.

[jlcd70112-bib-0043] Tarvainen, S. , S. Stolt , and K. Launonen . 2020. “Oral Language Comprehension Interventions in 1–8‐Year‐Old Children With Language Disorders or Difficulties: A Systematic Scoping Review.” Autism & Developmental Language Impairments 5: 239694152094699. 10.1177/2396941520946999. PMC9620463 36381544

[jlcd70112-bib-0044] Thompson, C. , D. McCaughan , N. Cullum , T. Sheldon , and P. Raynor. 2002. “The Value of Research in Clinical Decision Making.” Nursing Times 98, no. 42: 30–34. 12432662

