# Supplementary material for: 'Why Aren't We Fighting Our Case?': Speech and Language Therapists’ Perspectives on Intervention for Preschool Children With Oral Comprehension Difficulties
Source: Int J Lang Commun Disord. 2025 Aug 19;60(5):e70112. doi: 10.1111/1460-6984.70112 (PMC12362052; doi:10.1111/1460-6984.70112)
Supplement: Supplementary file 1 — Supporting Table S1: Description of intervention approaches, techniques and resources named by participants [file JLCD-60-0-s001.docx]

# **Supplementary material**

Table S1: Description of intervention approaches, techniques and resources named by participants

| Intervention approach | Description |
| --- | --- |
| Black Sheep Press | Organisation providing pictorial resources to support a range of children’s speech, language and communication needs. |
| Colourful Semantics | Intervention programme developed by Bryan (2003) which uses colour coding to teach children to identify thematic roles within spoken and/or written sentences, and use this knowledge to support the oral/written production of sentences using specific argument structures. |
| Derbyshire Language Scheme (DLS) | Language intervention programme developed by Knowles & Masidlover (1982). In the early stages of the programme, oral language comprehension and expression are developed through a structured progression of stages or ‘word levels’, which describe the number of ‘key words’ in an utterance the child can understand and use. |
| VERVE Child Interaction | Intervention programme developed by Cummins (n.d.), which aims to develop child language through enhancing parent/child interaction. |

**References (Supplementary material)**

Bryan, A. (2003). Colourful Semantics: Thematic Role Therapy. In A. Bryan, S. Chiat, J. Marshall, & J. Law (Eds.), *Language Disorders in Children and Adults* (pp. 143–161). Whurr.

Cummins, K. (n.d.). *VERVE Child Interaction*. Retrieved October 25, 2024, from http://www.keenacummins.co.uk/verve_child_interaction.html

Knowles, W., & Masidlover, M. (1982) *Derbyshire language scheme.* Distributed by Medoc Computer Ltd.
